# Combining a multi-analyzer stage with a two-dimensional detector for high-resolution powder X-ray diffraction: correcting the angular scale

**DOI:** 10.1107/S1600576721005288

**Published:** 2021-07-07

**Authors:** Andrew Fitch, Catherine Dejoie

**Affiliations:** a ESRF, 71 Avenue des Martyrs, CS40220, 38043 Grenoble Cedex 9, France

**Keywords:** high-resolution powder X-ray diffraction, analyzer crystals, two-dimensional detectors, axial divergence

## Abstract

In a test experiment, a two-dimensional pixel detector was mounted on the nine-channel multi-analyzer stage of the high-resolution powder diffraction beamline ID22 at the ESRF. This detector replaces a bank of scintillation counters that detect the diffracted intensity passing via the analyzer crystals as the diffractometer arm is scanned. At each diffractometer detector arm angle 2Θ, a 2D image is recorded that displays nine distinct regions of interest corresponding to the diffraction signals transmitted by each of the analyzer crystals. Summing pixels from within each region of interest allows the diffracted intensity to be extracted for each channel. X-rays are diffracted from the sample at various angles, 2θ, into Debye–Scherrer cones. Depending on the azimuthal angle around the cone, diffracted photons satisfy the analyzer-crystal Bragg condition at different diffractometer 2Θ values and arrive on the detector at different horizontal (axial) positions. The more the azimuthal angle deviates from diffraction in the vertical plane, the lower the 2Θ angle at which it is transmitted by an analyzer crystal, and the greater the distance of the detecting pixel from the centerline of the detector. This paper illustrates how the axial resolution afforded by the pixel detector can be used to correct the apparent diffraction angle, 2Θ, given by the diffractometer arm to its true diffraction angle, 2θ. This allows a reduction in peak asymmetry at low angle, and even with a relatively small axial acceptance, the correction leads to narrower peaks than if no correction is applied. By varying axial acceptance with diffraction angle, it is possible to optimize angular resolution at low diffraction angles and counting statistics at high angles. In addition, there is an intrinsic peak broadening with increasing azimuthal angle, dependent on the axial beam and detector pixel sizes. This effect reduces with 2θ, as the curvature of the Debye–Scherrer cones decreases. This broadening can be estimated and used to help choose the axial range to include as a function of diffraction angle.

## Abstract 2

The correction of the angular scale of powder diffraction data resolved axially by a two-dimensional pixel detector after an analyzer crystal or multi-analyzer stage is discussed. Angular correction reduces peak asymmetry at low angles and can be combined with a scheme that varies axial acceptance with angle to optimize angular resolution or statistical quality as required.

## 1. Introduction   

Dejoie *et al.* (2018 ▸) describe an experiment in which the scintillation counters behind the nine-channel multi-analyzer stage (Hodeau *et al.*, 1998 ▸) of the ID22 high-resolution powder diffraction beamline at the ESRF were replaced with a two-dimensional Pilatus3 X CdTe 300K-W pixel detector lent to us by Dectris. With the pixel detector, at each diffractometer detector arm angle, 2Θ, an image is recorded that displays nine distinct regions of interest corresponding to the diffraction signals transmitted via each of the analyzer crystals. Summing pixels from within each region of interest allows the diffracted intensity to be extracted for each channel.

X-rays are diffracted from the sample at various angles, 2θ, into Debye–Scherrer cones. Depending on the azimuthal angle around the cone, diffracted photons satisfy the analyzer-crystal Bragg condition at different diffractometer 2Θ values and arrive on the detector at different axial positions. The more the azimuthal angle deviates from diffraction in the vertical plane, the greater the axial divergence of a photon from the straight-through beam direction, the lower the 2Θ angle at which it is transmitted by an analyzer crystal and the greater the distance of the detecting pixel from the centerline of the detector. This effect is the cause of the low-angle peak-shape asymmetry seen in powder diffraction patterns and occurs whether an analyzer crystal is present or not, as an axially diverging photon will be intercepted by a receiving slit or position-sensitive detector at a lower diffractometer angle than a photon scattered in the vertical plane. Thus the greater the axial acceptance of a detector system, the greater the peak-shape asymmetry at low diffraction angles.

The use of a pixel detector has a number of advantages (Dejoie *et al.*, 2018 ▸), allowing considerable flexibility to optimize peak shapes and/or counting statistics via choice of the integration box width, reduction in parasitic scattering by careful choice of the area to be integrated, filtering of very bright pixels produced by large grains or single crystals in the sample thus improving the powder average, and the conferment on the measurement of a degree of depth resolution through the sample. In particular, by controlling the horizontal dimension of the integration box, asymmetry due to axial divergence could be reduced at low diffraction angles, leading to peak shapes that are more symmetric with an improvement in overall angular resolution. At higher diffraction angles, where the asymmetry effect is less because of the lower curvature of the Debye–Scherrer cones, a wider integration box could be exploited to improve counting statistics.

For a second test experiment, conducted around a year after the first, Dectris lent us the same type of detector, and further measurements were carried out. In this series of measurements, we were able to mount the detector more centrally (in the axial sense) on the arm of the diffractometer. The full horizontal acceptance of the detector was still shielded by other elements on the detector arm, but nevertheless we were able to collect data in the horizontal range from approximately −10.9 to 9.0 mm (pixel columns 45−160, covering 19.952 mm) either side of the centerline (pixel column ∼108).

In the processing of these data, rather than simply limiting the horizontal size of the integration box to influence the low-angle peak shape and high-angle counting statistics, we use the distance of each detecting pixel from the centerline of the pixel detector to correct the apparent diffraction angle, 2Θ, given by the mechanical position of the diffractometer arm to the true angle of diffraction, 2θ, defined by diffraction by the sample. The development builds on the work of Finger *et al.* (1994 ▸) and Ida *et al.* (2001 ▸), both of whom derive descriptions of low-angle asymmetry in a powder diffraction pattern collected with an analyzer crystal. We derive the correction to be applied to the diffractometer mechanical 2Θ value as a function of axial detection distance for an imperfectly aligned analyzer crystal in a multi-analyzer stage, suffering from misalignment about both the beam direction (crystal roll) and the vertical axis (crystal yaw). We incorporate the model into the Rietveld refinement program *TOPAS 6* (Coelho, 2018 ▸), and refine the parameters defining the geometry of the multi-analyzer stage from 1044 diffraction patterns of LaB_6_, corresponding to 116 patterns (pixel columns 45–160) for nine analyzer channels. The refined parameters are used to correct all the diffraction patterns to the true 2θ scale, and they can then be combined to produce a final diffraction pattern.

## 2. Experimental   

**Figure 1:**
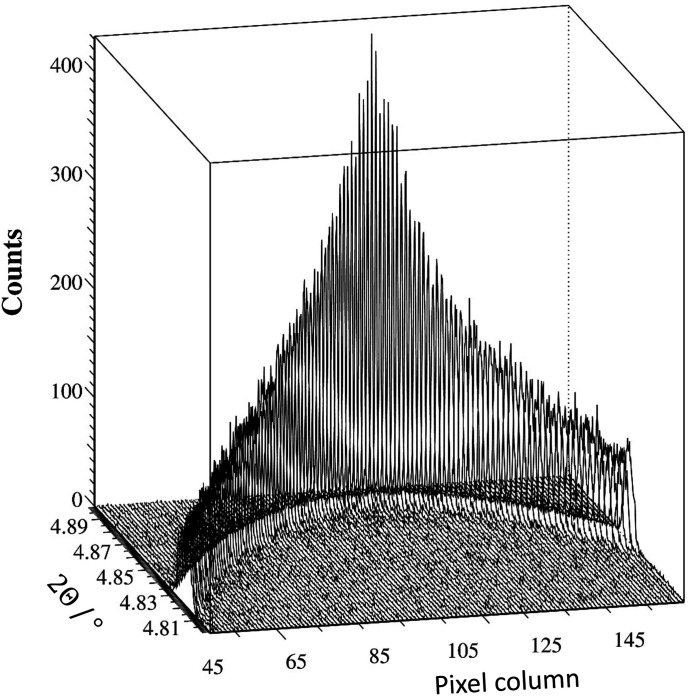
A set of 116 diffraction patterns showing the LaB_6_ 100 peak versus the detector pixel columns 45–160, corresponding to horizontal positions on the detector of −10.9–9.0 mm. The further from the centerline, the lower the diffractometer angle 2Θ at which the peak is transmitted by the analyzer crystal, and the broader the peak (though the integrated intensity does not change significantly).

The arrangement was as described by Dejoie *et al.* (2018 ▸), with the Pilatus3 X CdTe 300K-W pixel detector replacing the bank of standard scintillation counters behind the nine-channel multi-analyzer stage on ID22. A powder diffraction pattern was collected of NIST standard 660b, LaB_6_, at an energy of 35 keV, one of the convenient energies frequently used at the beamline. The principal difference from the previous experiment was the better axial centering of the detector on the arm of the diffractometer. The detector arm was scanned continuously at 2° per minute, collecting images at a rate of 66.67 Hz, so each image was recorded over an angular range of 0.0005° 2Θ. The full image size in pixels is 1475 (vertical) × 195 (horizontal), corresponding to 257 × 33.5 mm (pixel size of 172 × 172 µm). The nine regions of interest can easily be identified by adding all images together, so that all signals passing via a particular analyzer crystal superimpose. For each region of interest, at every angular position 2Θ, 116 contiguous zones across the detector, comprising 15 pixels vertical × 1 pixel horizontal, were used to obtain the number of counts recorded via that analyzer crystal, at that particular angle, at that particular horizontal position. The patterns collected via the central analyzer crystal over the 100 peak are shown in Fig. 1 ▸. Overall, 1044 diffraction patterns were extracted, which were used in *TOPAS 6* (Coelho, 2018 ▸) up to 45° 2θ for the refinement of the parameters characterizing the multi-analyzer stage.

## 3. Correcting the angular scale   

Finger *et al.* (1994 ▸) and Ida *et al.* (2001 ▸) derive expressions to describe the low-angle asymmetry of a powder diffraction pattern collected with an analyzer crystal. The first paper is mainly concerned with Debye–Scherrer geometry (without an analyzer crystal), but equation (A1) in the appendix gives an expression for the case of an analyzer crystal. The second paper considers an imperfectly aligned analyzer crystal with an element of roll of the crystal about the incident beam direction. Neither of these papers considers the effect of axial resolution, and expressions are obtained by integration over the axial receiving aperture. Ida (2020 ▸) also considers the equatorial aberration for an Si strip position-sensitive detector mounted in Bragg–Brentano geometry, a common configuration for modern laboratory diffractometers. In the present paper, we will follow the approach of Ida *et al.* (2001 ▸) but will also consider axial resolution of the signals via the pixel detector and allow an additional rotational aberration of the analyzer crystal about a vertical axis. This is because the crystals making up ID22’s multi-analyzer stage are attached to their support with high-temperature vacuum grease (to minimize strain), so there is scope for both types of rotational aberration. In the analysis, we find that the second rotation is unnecessary for fitting our observed diffraction patterns.

### 3.1. Single analyzer crystal   

**Figure 2:**
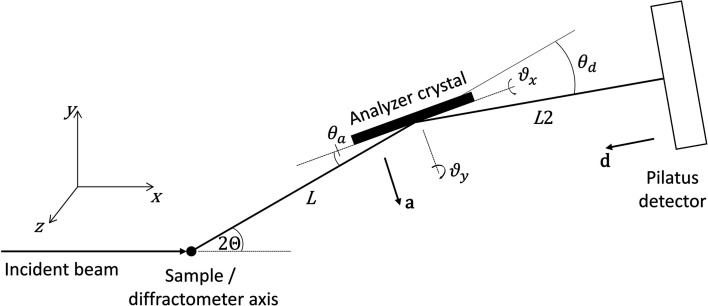
Schematic illustration of the mechanical setup with a single analyzer crystal. 2Θ is the angle of the diffractometer arm, θ_a_ the Bragg angle of the analyzer crystal, θ_d_ the rotation of the detector about the analyzer axis (typically twice θ_a_), **a** a unit vector normal to the analyzer and **d** a unit vector normal to the detector. *L* and *L*2 are the nominal distances between the diffractometer axis and the analyzer crystal, and between the analyzer crystal and the detector, respectively. With 2Θ = θ_a_ = 0, ϑ*_x_* is the rotation of the analyzer crystal about the **x** direction (roll aberration) and ϑ*_y_* the subsequent rotation about the **y** direction (yaw aberration). If ϑ*_x_* is zero, ϑ_y_ corresponds to a rotation about the crystal normal, so has no effect on diffraction.

We consider the case of a single analyzer crystal shown in Fig. 2 ▸.

#### 3.1.1. Diffraction at the analyzer crystal   

The unit vector normal to the analyzer crystal, **a**, is given by
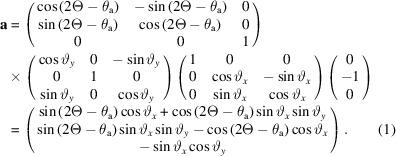
This is the same as equation (1) of Ida *et al.* (2001 ▸) if ϑ*_y_* is 0.

**Figure 3:**
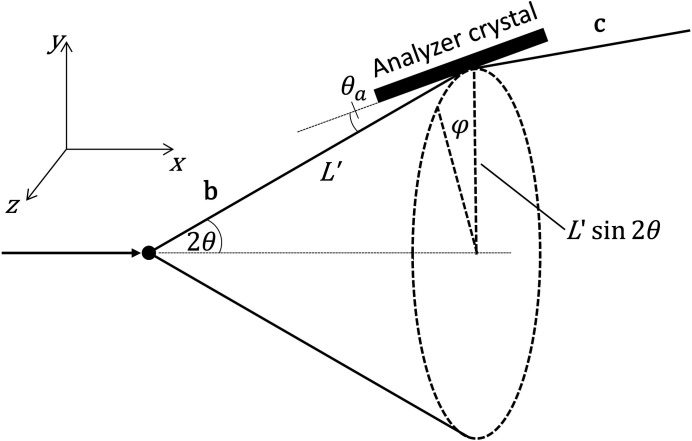
Diffraction by the sample into a Debye–Scherrer cone of half-angle 2θ (unit vector **b**) and then by the analyzer crystal (unit vector **c**).

The beam is diffracted by the sample by an angle of 2θ into Debye–Scherrer cones. The unit vector of the diffracted beam, **b**, is given by equation (2) of Ida *et al.* (2001 ▸) as
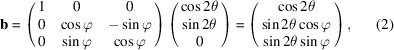
where φ is the azimuthal angle around the Debye–Scherrer cone, with φ = 0 taken as diffraction upwards in the vertical plane (Fig. 3 ▸).

**Figure 4:**
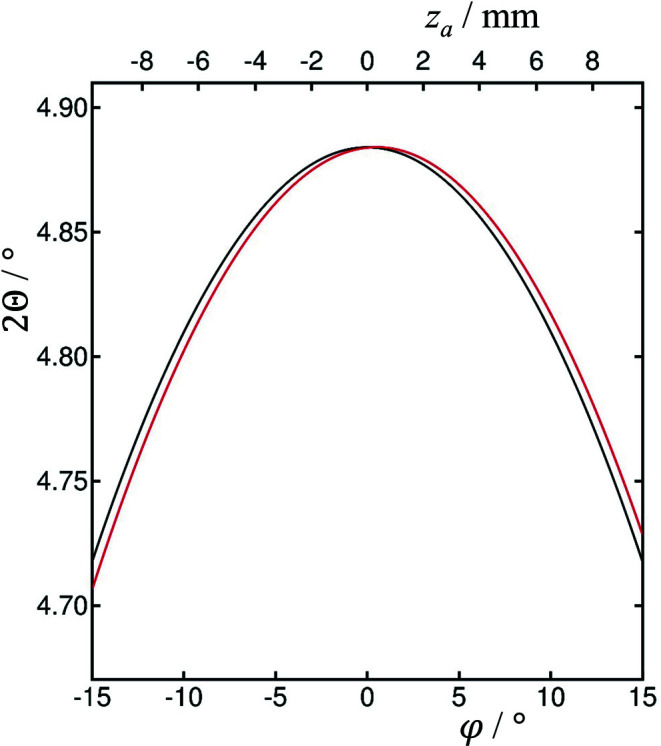
Calculated diffractometer arm angle, 2Θ, for the LaB_6_ 100 peak, *d* = 4.15689 Å, at 35 keV, 2θ = 4.88410°, as a function of φ, for ϑ*_x_* = 0 (black) and ϑ*_x_* = 0.5° (red). Also indicated is the corresponding axial distance, *z*_a_, of the diffracted beam on the Si 111 analyzer crystal (with *L* = 442.5 mm). Only when ϑ*_x_* = 0 is the curve symmetric about the vertical diffraction plane φ = 0. When ϑ*_x_* = 0.5° the maximum 2Θ is at 4.88416° at φ = 0.5002° with *z*_a_ = 0.328 mm.

To satisfy the Bragg condition at the analyzer crystal, the scalar product 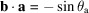 so that
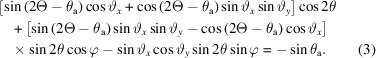
Fig. 4 ▸ illustrates how 2θ varies with φ for the LaB_6_ 100 reflection measured at 35 keV (2θ = 4.88410°).

**Figure 5:**
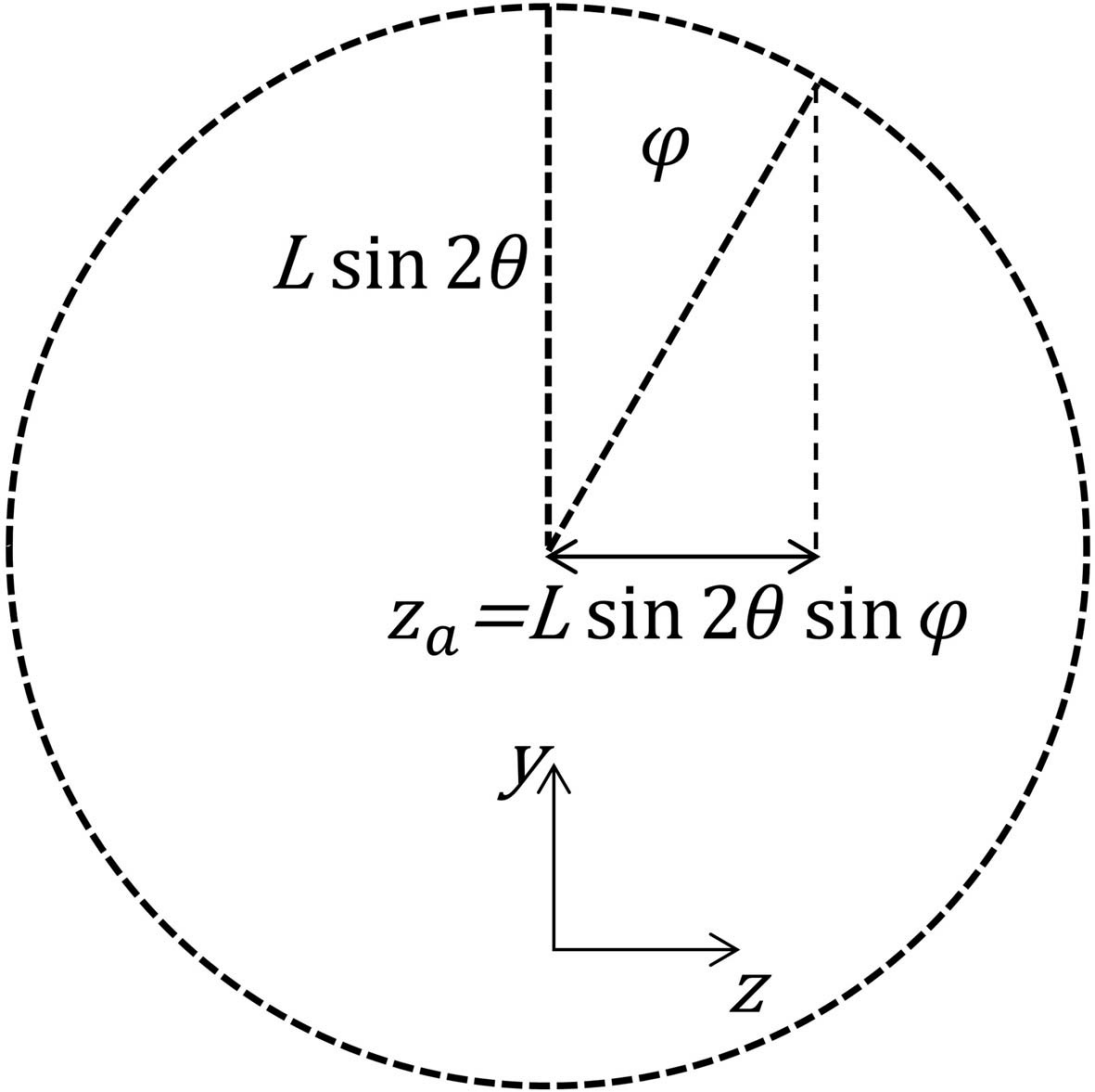
View along the axis of the Debye–Scherrer cone (*x* axis), showing the axial position, *z*_a_, where the diffracted beam is intercepted by the analyzer crystal.

The plane of the analyzer crystal is [via equation (1)]
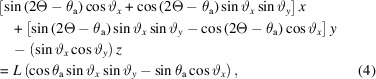
where *L* is the nominal distance along **x** from the sample on the diffractometer axis to the analyzer crystal at 2Θ = 0. If ϑ*_x_* is not zero, the distance, *L*′, to the point where the diffracted photon is intercepted by the analyzer [*L*′**b** = (*x*_a_, *y*_a_, *z*_a_), with 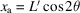, 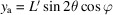 and 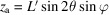] differs from *L*. From (4)

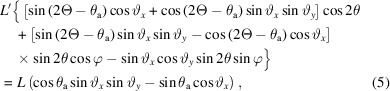


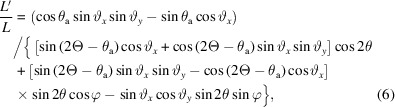
which simplifies via equation (3) to
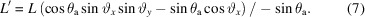
For an analyzer crystal that is well aligned, *L*′ ≃ *L*. If ϑ*_x_* is zero, *L*′ = *L* and 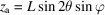 (Fig. 5 ▸), so that 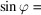 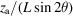 and 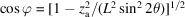. By introducing the latter into equation (3) with ϑ*_x_* set to zero, it is possible to obtain equation (A1) of Finger *et al.* (1994 ▸) (where *h* replaces *z*_a_); equation (A2) can be obtained from (3) above or equation (5) of Ida *et al.* (2001 ▸) with ϑ*_x_* zero.

#### 3.1.2. Distance to the detector   

The beam after diffraction (reflection) by the analyzer crystal, unit vector **c**, is given by
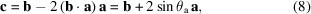


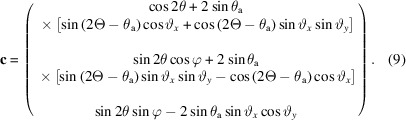
The pixel detector is normally mounted perpendicular to the straight-through beam and then rotated about the axis of the analyzer stage by θ_d_, usually twice the analyzer Bragg angle, so as to keep it perpendicular to the beam transmitted by the crystal (Fig. 2 ▸). (This condition is only possible for one crystal of a multi-analyzer arrangement.) The unit vector normal to the detector, **d**, after rotation of the diffractometer by 2Θ is given by
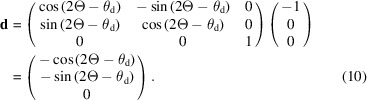
θ_d_ should account for any offset due to mounting misalignment or zero error on the stage axis, or can even be chosen so as to spread the diffracted beam over a greater number of pixels along the length of the detector to improve the depth resolution in the sample that a pixel detector confers (Dejoie *et al.*, 2018 ▸). With a distance *L*2 along **d** between the analyzer crystal and the detector, the plane of the detector is
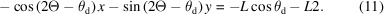
The distance traveled by the diffracted beam from the analyzer crystal to the detector varies with φ and is designated as *L*3. The diffracted ray arrives at the detector at point (*x*_d_, *y*_d_, *z*_d_) given by *L*′**b** + *L*3**c**, so
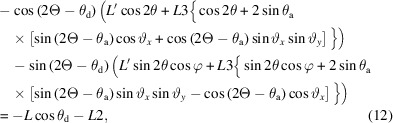


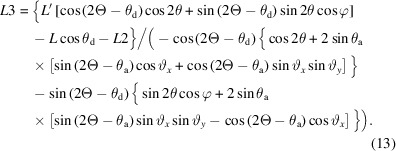



#### 3.1.3. Calculating the shift in peak position   

Axially, from (2) and (9),
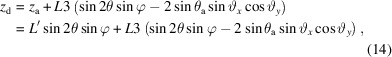


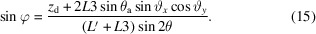
Equation (15) allows φ to be obtained from the axial position of the receiving pixel, *z*_d_. From an approximate starting value for φ, *e.g.* 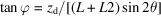, which is obtained by extrapolation of equation (3) of Ida *et al.* (2001 ▸) (though this is only really valid up to the analyzer crystal), equation (3) above allows calculation of the diffractometer angle, 2Θ, at which the analyzer crystal transmits the beam; equation (13) then gives the corresponding value of *L*3, and equation (15) provides a new estimate of φ. The process is iterated and converges in a few cycles. Equation (3) is exploited by rearranging it to
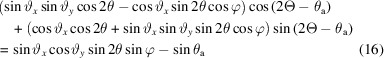
or
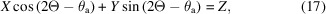


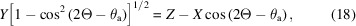


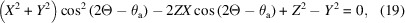


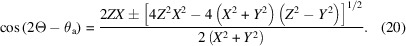
The ‘+’ solution to this quadratic is the appropriate one for angles of 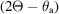 up to 90°. The value of 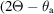) is obtained by taking cos^−1^ of the right-hand side of (20). A small modification at low angles near 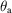 and below is described in the supporting information.

**Figure 6:**
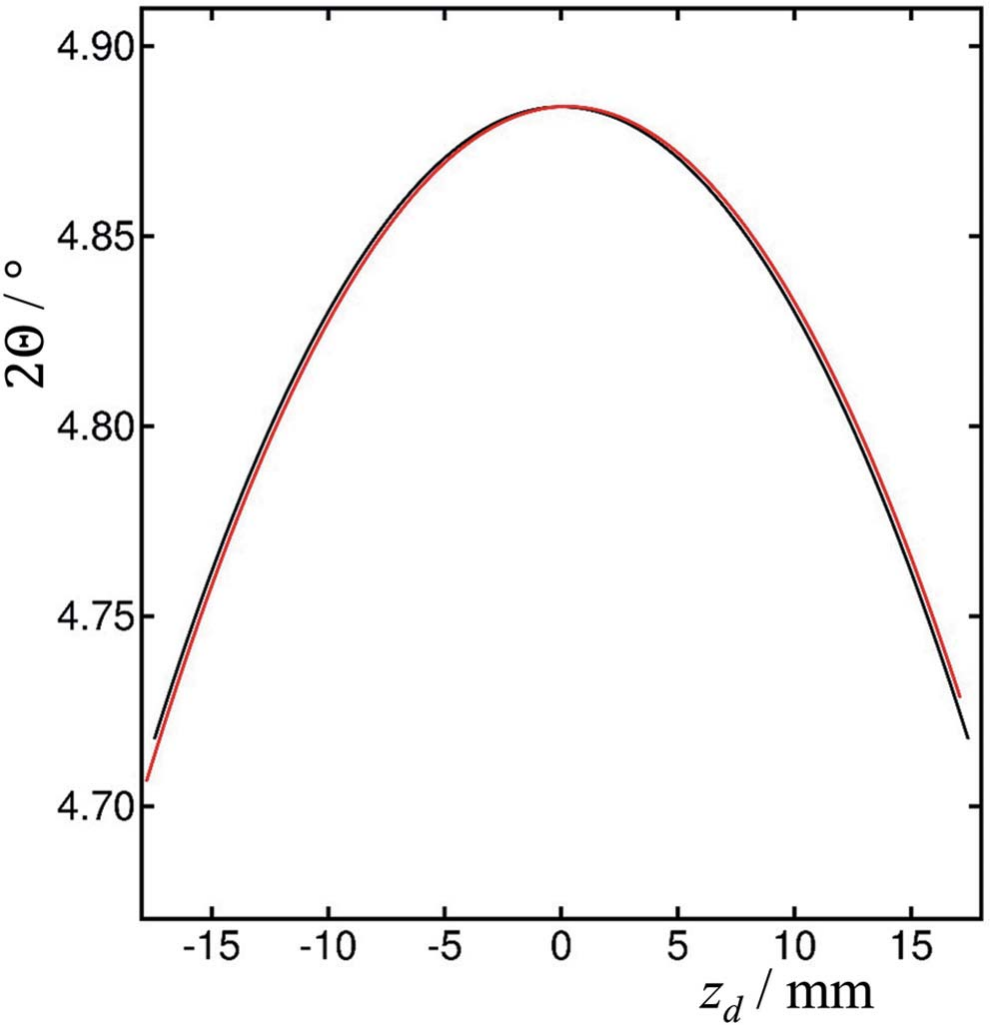
Calculated diffractometer arm angle, 2Θ, in degrees for the LaB_6_ 100 peak, *d* = 4.15689 Å, at 35 keV, 2θ = 4.88410°, as a function of *z*_d_, with *L* = 442.5 mm, *L*2 = 350 mm, θ_a_ = 3.2382° (Si 111 analyzer), for ϑ*_x_* = 0 (black) and ϑ*_x_* = 0.5° (red). The analyzer roll results in the maximum of the curve moving to 2Θ = 4.88416° at φ = 0.5002°, with *z*_d_ = 0.243 mm, and the corresponding *z*_a_ = 0.328 mm, as the roll deflects the beam back towards the center.

The calculated 2Θ positions for the LaB_6_ 100 peak at 35 keV as a function of *z*_d_ are shown in Fig. 6 ▸, for ϑ*_x_* = 0 and ϑ*_x_* = 0.5°, as for Fig. 4 ▸. The analyzer roll results in the maximum of the curve moving from 2Θ = 4.88410° to 2Θ = 4.88416° at φ = 0.5002°, with *z*_d_ = 0.243 mm, and the corresponding *z*_a_ = 0.328 mm, as the roll of the analyzer crystal deflects the axially diverging beam back towards the center of the detector.

Experimentally, owing to the horizontal beam and pixel sizes, each pixel detects X-rays encompassing a range of *z*_d_ values, *z*_d_ ± δ*z*_d_. This means that for each pixel, at distance *z*_d_, there is a range in the corresponding φ and 2Θ values contributing to that position, an intrinsic uncertainty in the angular range received by that pixel, resulting in an additional term influencing the width of a diffraction peak. The shift in apparent peak position accelerates with the magnitude of *z*_d_, so peak widths increase with the distance from the centerline of the detector, dependent on the difference in angular correction for *z*_d_ and *z*_d_ ± *δz*_d_.

**Figure 7:**
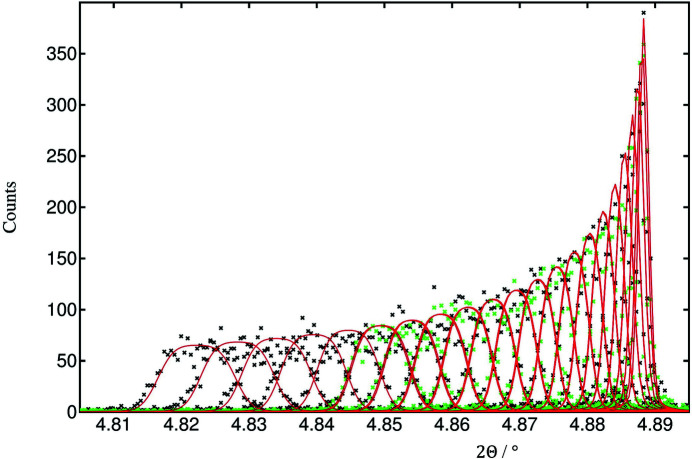
Observed (points) and calculated (red curve) diffraction patterns, after Rietveld refinement over the full angular range, showing the fit to the LaB_6_ 100 peak for the central channel of ID22’s multi-analyzer stage. Black points correspond to negative *z*_d_ values (pixel columns 45–108) and green points to positive *z*_d_ values (pixel columns 109–160). For clarity, only every third pattern is shown.

The above scheme to calculate the shifts in peak positions and intrinsic broadening (the latter as the difference of the shift in peak position for *z*_d_ and *z*_d_ ± *δz*_d_, scaled by two refinable parameters, one for a Gaussian contribution, the other for a double stacked_hats contribution) was included in a Rietveld refinement using *TOPAS 6* to fit the series of patterns from the LaB_6_ standard corresponding to different *z*_d_ values across the detector. Nine iterations of equations (3), (13) and (15) were coded into each refinement cycle. The differences between the true 2θ values, calculated from the lattice parameter of the sample, and the diffractometer 2Θ angle at which a reflection is transmitted by the analyzer crystal (dependent on 2θ and *z*_d_) were included in the peak-position offset parameter (th2_of fset). In such a scheme, any of the usual quantities such as wavelength and diffractometer zero-point correction could be refined, and also parameters relevant to the analyzer stage, such as *L*2, ϑ*_x_, ϑ_y_*, the zero position of *z*_d_ and even θ_d_ (though that should normally be known and fixed). Note that the diffractometer zero point and θ_a_ are 100% correlated as they equally affect the diffractometer angle at which the analyzer crystal transmits the diffracted beam. Perhaps surprisingly, ϑ*_y_* was also found to be 100% correlated with zero point. Although it can significantly affect the correction to the angular scale, the effect appears essentially constant with 2θ and φ, so ϑ*_y_* can be set to zero without affecting the quality of the refinement. The Rietveld fit to the LaB_6_ patterns, with fixed atomic parameters, collected via the central crystal on the axis of rotation of ID22’s multi-analyzer stage is illustrated for the LaB_6_ 100 peak in Fig. 7 ▸. Any standard with known lattice parameters, such as Si, could be used in place of LaB_6_.

Once the parameters defining the analyzer crystal have been refined, they can be used to correct the 2Θ angular scale on which an individual data set has been measured to the true 2θ scale to which the recorded intensities correspond.

**Figure 8:**
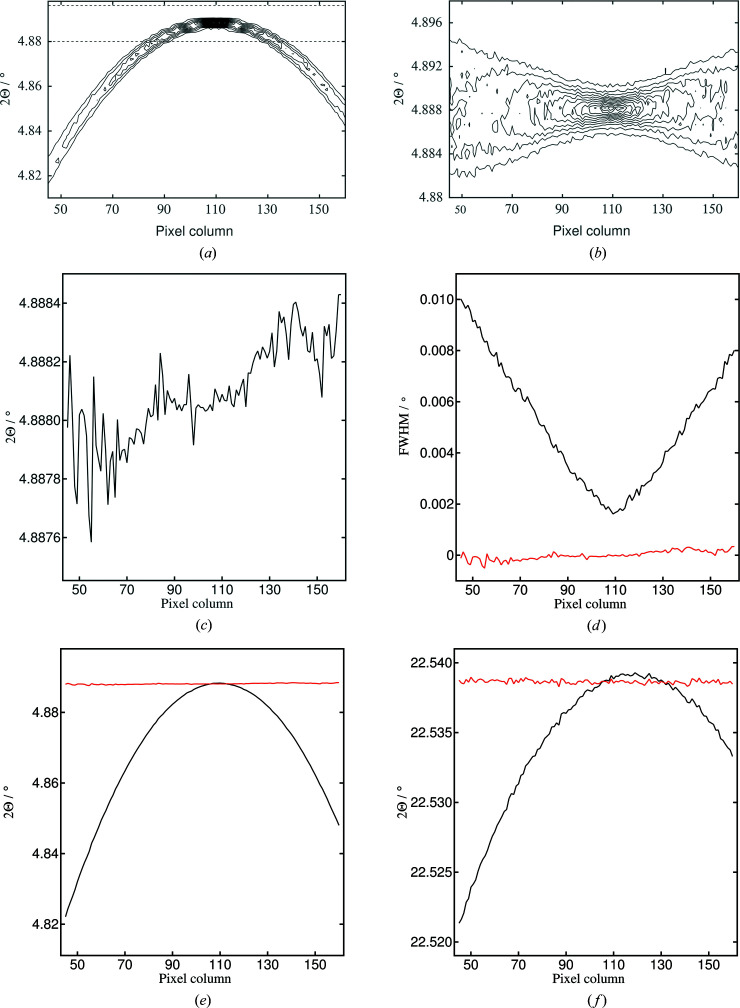
(Top) Contour plots of 2Θ versus pixel column as the detector arm is scanned through the LaB_6_ 100 peak: (*a*) the raw data (*cf*. Fig. 1 ▸), with the horizontal dashed lines indicating the angular range shown in (*b*), corresponding to the values after correction. (Middle) Fit of a simple pseudo-Voigt function to the corrected values, showing (*c*) the fitted positions and (*d*) the FWHM and, in red on the same scale, the difference between the fitted position and the average fitted position. The corrected positions cover a very small angular range compared with the FWHM values, though there are perhaps indications of a residual systematic trend. (Bottom) Fitted positions of the pseudo-Voigt function (*e*) to the 100 peak (raw data in black and corrected data in red) and (*f*) to the 421 peak. Note that the maximum in peak position for the raw data moves axially with angle because of the roll of the crystal.

From (3),
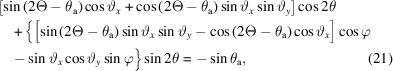

*i.e.*

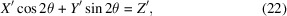
so 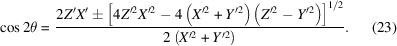
Again the process is iterative, starting from an approximate φ, *e.g.* 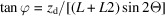, using (23) to obtain 2θ, then (13) for *L*3, then an updated φ from (15). Some results from doing this for the LaB_6_ 100 peak are shown in Fig. 8 ▸.

The effectiveness of the correction of the angular scale can be seen in comparing the intensity contour plots shown in Figs. 8 ▸(*a*) and 8 ▸(*b*). Fitting a simple pseudo-Voigt function to the corrected data shows an average position for the 100 peak of 4.88809 (17)°, with values covering a range of 0.00085°, which can be compared with a spread in peak positions of 0.066° in the uncorrected data. The widths of the peaks [Fig. 8 ▸(*d*)] show a clear minimum towards the centerline of the detector as expected. The fitted peak positions, however, show a clear trend across the detector, which was not expected. Ideally there should be no such trend. We believe this is due to residual misalignment in the system not taken into account in the current model. If, for example, the whole diffractometer is allowed to rotate about the vertical (*y*) axis, then this modifies the axial position of the diffracted beam arriving on the detector and hence the value of *z*_d_, which is 2Θ dependent. Introducing a rotation of 30 µrad (0.0017°) about the vertical axis into the model leads to the plot shown in Fig. S2, where the systematic trend is essentially eradicated. Thus, in principle, such overall alignment parameters might also be extracted from the axially resolved powder diffraction data. We have not done this systematically here as the quality of the data from this test experiment is not sufficient to extract such subtle quantities reliably. In Figs. 8 ▸(*e*) and 8 ▸(*f*), the fitted positions to the uncorrected and corrected peak positions are shown on the same scale, for the 100 and 421 peaks. The maximum correction in peak position is 0.066° for the 100 peak and 0.017° for the 421 peak, for which, being at higher angle, the curvature of the Debye–Scherrer cone is lower. The effect of the roll of the crystal can be seen by the displacement with angle in the axial position of the maxima of the two curves, and also the correction of 2Θ to lower values near the maxima.

## 4. Multi-analyzer stage   

The ID22 multi-analyzer stage (Hodeau *et al.*, 1998 ▸), conceived for the original ESRF powder diffraction beamline (BM16), consists of nine Si 111 crystals mounted on a rotation stage, each separated from its neighbor by nominally 2°. The channels are numbered 0−8, with the central channel 4 mounted on the rotation axis of the stage which is taken as corresponding to the diffractometer mechanical angle 2Θ. The crystals are set on a radius of 442.5 mm, *L*, the distance between the diffractometer and multi-analyzer axes. For the central crystal, this distance, *L*_4_ = *L*, does not change when the Bragg angle of the crystal, θ_a_, is set. All the other crystals move spatially, and the distance from the sample to the crystal, *L_n_*, becomes
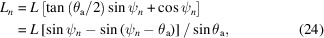
where ψ*_n_* is the offset between channel *n* and the central channel (see supplementary information). Equation (3) becomes
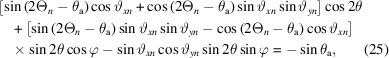
where 2Θ*_n_* = 2Θ + ψ*_n_*.

**Figure 9:**
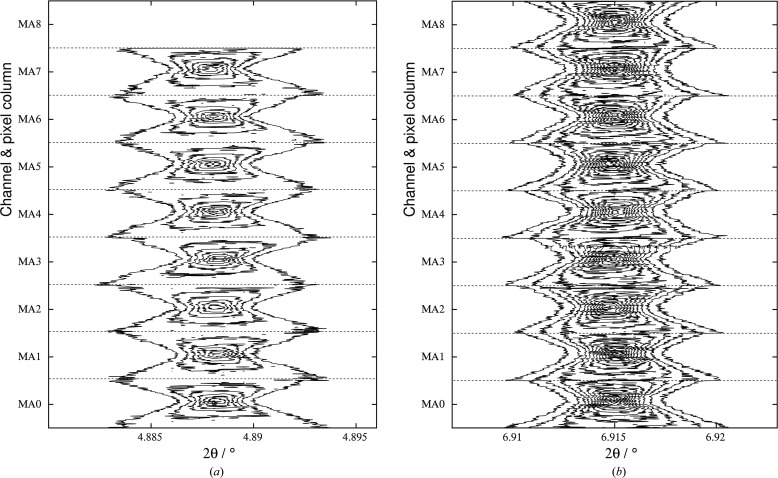
Contour plots of intensity versus angle after correction for the nine detector channels and 116 pixel columns (45–160) for LaB_6_: (*a*) the 100 peak and (*b*) the 110 peak. Starting the data collection from −3° 2Θ means that we narrowly missed 100 with MA8, which detects at an angle of 2Θ + ∼8°. For each channel, the peaks go from broad to narrow to broad again as we move across the detector (corresponding to the vertical direction of the plot for each channel shown).

Each crystal can be considered in the same way as a single analyzer with distance *L_n_*, so by analogy with (7), the distance traveled by a photon diffracted by angle 2θ from the sample to the crystal is
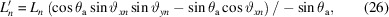
and, by analogy with (9), the path after diffraction from the crystal is
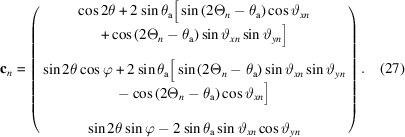
The diffracted X-ray beam arrives at the detector, defined by (11), at point (*x*_d*n*_, *y*_d*n*_, *z*_d*n*_) given by *L*′*_n_* **b** + *L*3*_n_* **c** *_n_*, so
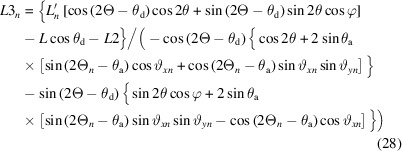
and
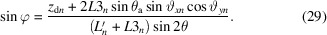
As for a single analyzer, from an initial estimate of φ, *e.g.* 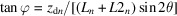, equation (25) allows calculation of the diffractometer angle at which the analyzer crystal transmits the beam, via
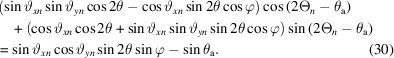
Equation (28) then gives the corresponding value of *L*3*_n_*, and equation (29) provides a new estimate of φ, *etc*. Incorporated into the Rietveld refinement (the *TOPAS* input file is provided as supporting information), data from all nine channels (116 horizontal positions per channel) can be fitted simultaneously. Additional parameters that can be refined include scale factor and offset ψ*_n_* for each channel (information needed for combining all the channels later), and the roll of the detector in case it is not mounted perfectly: a small rotation about the **x** direction would lead to a progressive change in the zero of *z*_d*n*_ between channels. The extracted parameters can then be used to correct the 2Θ angular scale depending on *z*_d*n*_ iteratively, as before for a single analyzer crystal, via equations (25),
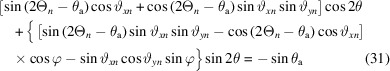
to obtain a new value of 2θ, (28) to calculate *L*3*_n_* and then (29) for an update of φ. The LaB_6_ 100 and 110 peaks so treated are shown in Fig. 9 ▸ for all nine channels.

## 5. Combining the nine channels   

The method of combining the counts from a continuous scan for the nine channels is described by Wright *et al.* (2003 ▸). In brief, the accurately calibrated angular offsets between channels (multiples of ∼2°) are applied when combining the counts from the different channels into bins of chosen angular step, taking into account the relative efficiency of each channel and any change in synchrotron intensity (monitor counts) during the measurement. Values recorded across bin boundaries are partitioned proportionally. Counts can be combined from multiple scans of the sample, including different parts of the diffraction pattern, *e.g.* when several scans have been made at higher diffraction angles to improve statistical quality, an approach often adopted for high-quality pair-distribution function analysis. The same overall procedure is applied here, although instead of there being nine detector signals to process, there are now 1044. A step size of 0.0003° appeared to be appropriate. If the angular-correction process were perfect, there would be no further angular offsets to apply between analyzer crystals. There are, however, minor residual discrepancies, and we allow the rebinning program to calibrate and apply a small average correction per crystal, the greatest in magnitude being −0.0016°.

There are many ways to choose the axial range to be included in the rebinning of the data. The simplest is to take the range of ±*s* mm either side of the centerline of the detector, analogous to the situation with a physical axial receiving slit. Alternatively, the axial acceptance can vary with 2θ, *e.g.* a linear dependence or sin2θ dependence, or anything else that might seem reasonable. Note that it is not necessary to throw data away (except at the lowest 2θ angles where the Debye–Scherrer ring is completely contained within the width of the detector), as a second rebinning of the data could be carried out to produce a second data set, of lower angular resolution, composed of the data not selected for the first. This could be included in a multi-pattern Rietveld refinement if appropriate. One way of ensuring that the highest-resolution data have been included, *e.g.* for indexing or space-group assignment, is to include only data whose nominal intrinsic broadening is less than a chosen value. This automatically increases the axial acceptance as 2θ increases (as the curvature of the Debye–Scherrer rings decreases) and also tracks the position of the data on the detector with the minimum intrinsic width, which evolves with angle because of the effect of crystal roll [*e.g.* see the effect by comparing the axial position of the maxima in peak position in Figs. 8 ▸(*e*) and 8 ▸(*f*)].

**Figure 10:**
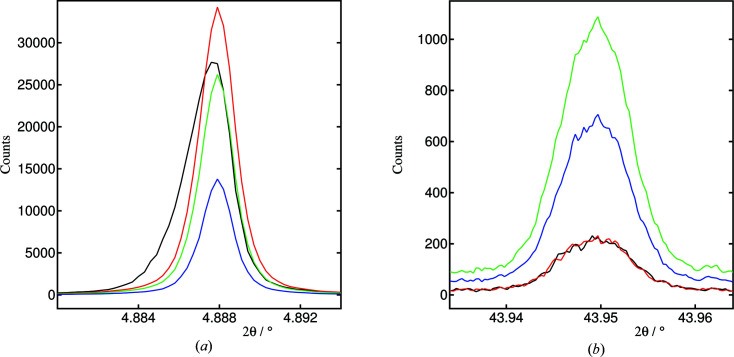
Plots showing the effect of binning in different ways: (*a*) 100 peak and (*b*) 654 peak; black, uncorrected data, 4 mm axial acceptance; red, angular scale corrected, 4 mm axial acceptance; green, corrected, intrinsic broadening ≤ 0.002°; blue, corrected, intrinsic broadening ≤ 0.001°.

**Figure 11:**
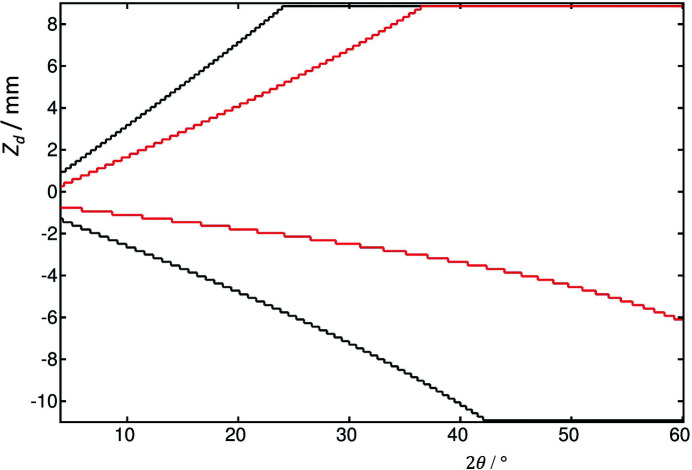
Limits of the z_d_ values accepted versus 2θ for a nominal intrinsic broadening of (black) ≤0.002° and (red) ≤0.001° for central channel 4. The differences across the width of the detector are due to the roll of the corresponding crystal, refined to 0.3498 (1)°

Examples of the LaB_6_ 100 and 654 peaks reconstructed by summing in steps of 0.0003° with various conditions are plotted in Fig. 10 ▸. Fig. 11 ▸ shows the extents of the axial ranges with angle for intrinsic broadening of ≤0.002 and ≤0.001°. Parameters obtained from fits to these peaks are given in Table S1. The peaks were fitted with a Voigt function, and possibly a *TOPAS* correction for asymmetry (Full_Axial_Model) or one or two exponential contributions (via the exp_conv_const command), which were found to improve agreement significantly. The exp_conv_const command leads to the inclusion of an exponential function into the overall convolution of Gaussian and Lorentzian functions that *TOPAS* performs in calculating the diffraction peak profile. If the associated parameter is negative, this modifies the peak profile on the low-angle side of the peak, and vice versa for a positive parameter.

It is apparent in the uncorrected data (4 mm fixed axial acceptance) that the 100 peak shows asymmetry and broadening [Fig. 10 ▸(*a*)]. Acceptable fits are obtained only when including asymmetric contributions to the peak shape, via the asymmetry correction, or, less effectively, one or two exponential contributions to the peak shape.

Correcting the angular scale to the true 2θ scale while keeping the same axial acceptance of 4 mm reduces the asymmetry and sharpens the peak. Also evident is the shift to higher angle of the peak profile intensity. Reducing the axial acceptance by requiring that the intrinsic broadening should be below a certain value reduces the peak intensity but leads to further sharpening of the peak. Fitting the Voigt function alone, the FWHM decreases from the raw data [0.00272 (3)°] to the angle-corrected data: 4 mm acceptance [0.00215 (1)°], intrinsic broadening ≤0.002° [0.00202 (1)°], intrinsic broadening ≤0.001° [0.00189 (1)°]. For the last two cases, the intensity is reduced because the axial range accepted across the detector at the 100 peak is now less than 4 mm (Fig. 11 ▸). For any particular experiment, the balance between intensity (statistical quality) and peak width will depend on the nature of the study.

For all fits (Table S1), including an asymmetry correction or one or two exponential functions improves the fit compared with a Voigt function alone, by as much as a factor 3.4 in goodness of fit (GOF) when using the Full_Axial_Model for the obviously asymmetric peak in the uncorrected data. For the corrected data, the peaks are less asymmetric. Nevertheless, some improvement is seen when including an asymmetry correction or exponentials, suggesting a small degree of residual asymmetry, although this is not easily perceptible by eye. This should perhaps be expected as the axial divergence is not completely eliminated; a 1.2 mm-wide beam at 800 mm from a 4 mm axial receiving range is replaced by a 1.2 mm beam and nominally a 0.172 mm receiving pixel, representing a decrease of axial divergence of about 74%. This implies that, because of the size of the incident beam, even a single pixel element receives X-rays diffracted through a small range of φ values with an accompanying intrinsic asymmetry effect. However, the refined values of the receiving aperture in the asymmetry correction are much larger than expected from this perspective, at around 2.6 (1) mm.

Because the asymmetry correction or exponentials affect the two sides of the peak differently, there are significant correlations between these parameters and the refined position of the peak (see Table S1). Hence a degree of variability is to be expected in the refined peak position depending on the fitted model. However, for the angle-corrected data there is consistency between the peak positions for data summed in different ways for each of the models. Whereas the variability of a peak’s refined position with model might be an issue for studies when refinement of a single peak’s position is important (such as in mapping residual strain in a component or surface), it will be less so in a Rietveld refinement where peak positions are defined by the refined lattice parameters and so are constrained relative to other peaks across a diffraction pattern.

For the 654 peak [Fig. 10 ▸(*b*)], correcting the angular scale of the data leads to only a small decrease in peak width, less than 10% between the uncorrected and intrinsic broadening ≤0.001° patterns, since the curvature of the Debye–Scherrer ring is much less at such angles. However, a significant increase in intensity can be realized, up to a factor of 4.7 compared with the 4 mm aperture, which is limited by the maximum axial acceptance of the current experimental setup. With a larger axial acceptance, intensities could be enhanced further. Also of note is that the fits to all patterns were improved significantly by including an essentially symmetric exponential component in the peak profiles.

If all the corrected data are integrated across the full ∼20 mm of the detector (all 116 active pixel columns) then there is a significant increase in the intensity of the 100 peak by a factor of ∼5. The peak is significantly broadened [FWHM of 0.00500 (4)°] because of the intrinsic broadening with increasing values of φ and *z*_d_. Nevertheless, an FWHM of 0.005° might be considered acceptable in some circumstances for a high-quality powder diffraction pattern, *e.g.* where sample broadening effects are present and microstructural information from peak-shape analysis is not the aim of the study, or for PDF analysis of poorly crystalline materials. The peak is best fitted by a symmetric peak shape (Fig. S3), including exponential components; refinements with an asymmetry correction or single exponential did not lead to satisfactory fits, with a GOF near 10.

## 6. Rietveld refinement   

**Figure 12:**
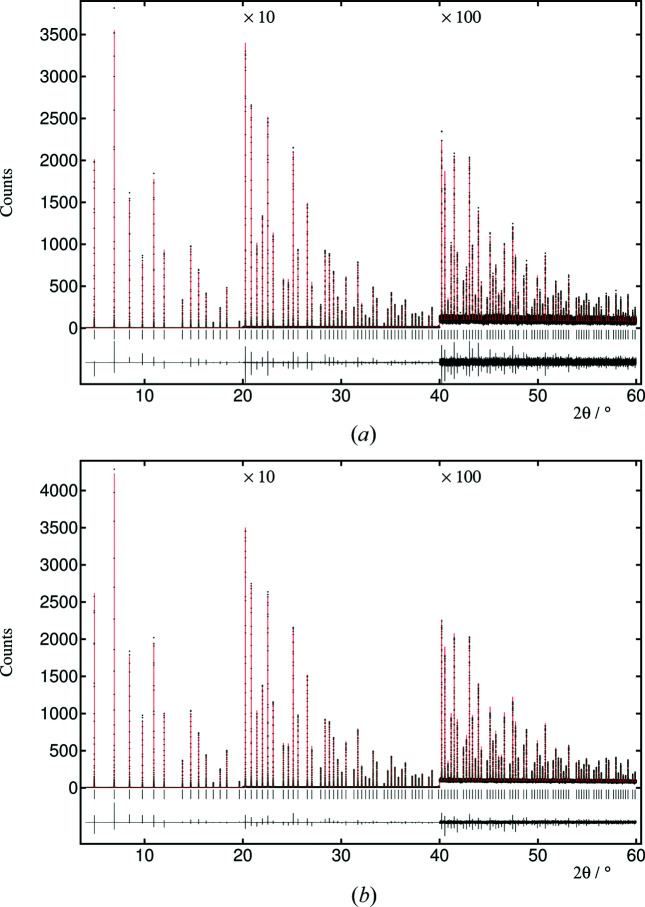
Observed (points), calculated (red line) and difference profiles calculated by *TOPAS* for renormalized (to equivalent monitor counts) data: (*a*) uncorrected data, 4 mm axial aperture, *R*_wp_ = 13.66, *R*_exp_ = 12.83, GOF = 1.06; (*b*) angle-corrected data, intrinsic broadening ≤ 0.002°, *R*_wp_ = 8.83, *R*_exp_ = 7.61, GOF = 1.16. Data and refinement files are provided as supporting information.

Rietveld refinements were performed in *TOPAS 6* using uncorrected and angle-corrected data sets. Peak shapes were described as a Voigt function with an asymmetry correction and additional asymmetric exponential contributions, varying as 1/(θ + ɛ) (where ɛ is an offset), an empirical contribution that improves the fits. Both patterns can be fitted satisfactorily, and fits are shown in Fig. 12 ▸. Nevertheless, it is clear that the statistical quality of the pattern obtained with an increase in axial acceptance with angle is better (*R*_exp_ = 7.61 versus *R*_exp_ = 12.83), particularly at higher diffraction angles, above ∼42°, where the axial acceptance of ∼20 mm is five times greater than when using a fixed width of 4 mm.

## 7. Conclusion   

Mounting a pixel detector behind an analyzer crystal or multi-analyzer stage can lead to an improvement in the angular resolution of the powder diffraction pattern by allowing the apparent shift in peak position with axial divergence of the diffracted beam from the vertical plane to be corrected to the true 2θ value defined by diffraction from the sample. The shift in peak position with axial divergence is the cause of the low-angle asymmetry in peak shapes seen in powder diffraction patterns. The axial divergence depends on the azimuthal angle around the Debye–Scherrer cone of the diffracted ray emanating from the sample. From the measurement of the diffraction pattern of a suitable standard such as LaB_6_, the parameters defining the geometry of the analyzer stage can be refined against data recorded as a function of distance from the centerline of the detector via the Rietveld method. Even imperfections in the mounting of the analyzer crystal(s), such as roll about the beam direction, can be characterized and their effects on apparent peak position taken into account.

There is an intrinsic broadening of the diffraction peaks with azimuthal angle and corresponding axial divergence from the vertical plane, owing to the curvature of the Debye–Scherrer cones. This broadening also depends on the axial sizes of the incident beam and the receiving pixel, which define a range of axial distances (or azimuthal angles) arriving on a particular pixel, and can be estimated and used to control the axial extent of the data to be included in the diffraction pattern. This allows the angular resolution to be tuned to the problem under investigation. Improvement in the statistical quality of the powder diffraction pattern is also possible by allowing the axial acceptance of the data to vary with diffraction angle. There are many ways that such schemes can be devised, but one dependent on the estimated intrinsic broadening allows a natural increase in the axial range of the data to be included with increasing diffraction angle, where count rates decrease as a result of the X-ray scattering form factor and Debye–Waller factors.

In the current study, using a Dectris Pilatus3 X CdTe 300K-W pixel detector which was temporarily mounted on the standard detector arm of the ID22 diffractometer, we were limited to a total axial range of ∼20 mm, because of the X-ray window sizes on the arm. In the future, we plan to install an Eiger2 X 2M-W CdTe detector as a permanent feature on a dedicated arm, with a smaller pixel size of 75 µm and a width of ∼38 mm. Correction of the angular scale as a function of axial divergence and variable axial acceptance will be implemented as standard options in the data reduction procedures.

## Supplementary Material

Supporting information file. DOI: 10.1107/S1600576721005288/kc5125sup1.pdf

Topas input file. DOI: 10.1107/S1600576721005288/kc5125sup2.txt

Topas xdd input file. DOI: 10.1107/S1600576721005288/kc5125sup3.txt

Rebinned xye data for Fig12a. DOI: 10.1107/S1600576721005288/kc5125sup4.txt

Topas input file for Fig 12a. DOI: 10.1107/S1600576721005288/kc5125sup5.txt

Rebinned and angle-corrected xye data for Fig 12b. DOI: 10.1107/S1600576721005288/kc5125sup6.txt

Topas input file for Fig 12b. DOI: 10.1107/S1600576721005288/kc5125sup7.txt

## References

[bb1] Coelho, A. A. (2018). *J. Appl. Cryst.* **51**, 210–218.

[bb2] Dejoie, C., Coduri, M., Petitdemange, S., Giacobbe, C., Covacci, E., Grimaldi, O., Autran, P.-O., Mogodi, M. W., Šišak Jung, D. & Fitch, A. N. (2018). *J. Appl. Cryst.* **51**, 1721–1733.

[bb3] Finger, L. W., Cox, D. E. & Jephcoat, A. P. (1994). *J. Appl. Cryst.* **27**, 892–900.

[bb4] Hodeau, J. L., Bordet, P., Anne, M., Prat, A., Fitch, A. N., Dooryhée, E., Vaughan, G. & Freund, A. (1998). *Proc. SPIE*, **3448**, 353–361.

[bb5] Ida, T. (2020). *J. Appl. Cryst.* **53**, 679–685. 10.1107/S1600576720005130 PMC7312143 32684883

[bb6] Ida, T., Hibino, H. & Toraya, H. (2001). *J. Appl. Cryst.* **34**, 144–151.

[bb7] Wright, J. P., Vaughan, G. B. M. & Fitch, A. N. (2003). *IUCr Commission on Crystallographic Computing Newsletter*, No. 1, pp. 92–96.

