# Supplementary material for: Combining a multi-analyzer stage with a two-dimensional detector for high-resolution powder X-ray diffraction: correcting the angular scale
Source: J Appl Crystallogr. 2021 Jul 7;54(Pt 4):1088–99. doi: 10.1107/S1600576721005288 (PMC8366427; doi:10.1107/S1600576721005288)
Supplement: Supplementary file 1 [file j-54-01088-sup1.pdf]

JOURNAL OF APPLIED CRYSTALLOGRAPHY

Volume 54 (2021)

Supporting information for article:

Combining a multi-analyzer stage with a 2-dimensional detector for high-resolution powder X-ray diffraction: correcting the angular scale

Andrew Fitch and Catherine Dejoie

S1. Multi-analyzer stage

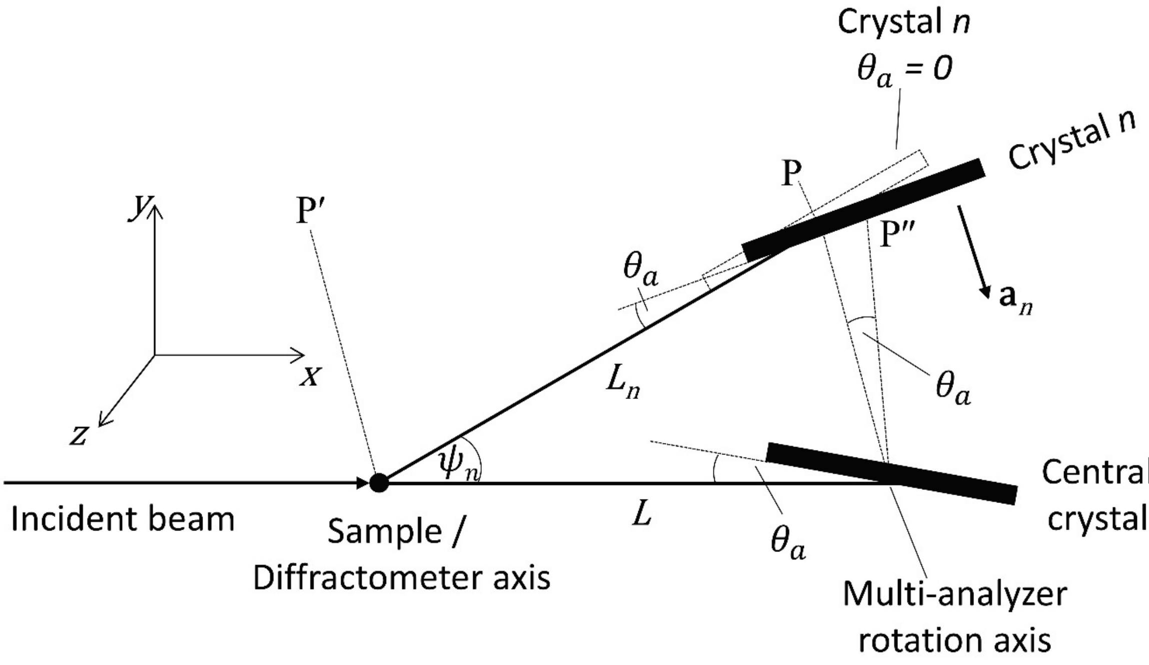

Figure S1 Schematic drawing of two crystals in a multi-analyzer stage. The central crystal is mounted on the axis of rotation of the multi-analyzer stage, at distance L from the diffractometer (sample) axis, with additional crystals at relative angles ψ_n. When the Bragg angle θ_a of the assembly is set, the central crystal rotates about the stage axis while all other crystals are displaced in space.

Consider a point P in the vertical plane z = 0 on the surface of crystal n at distance L from the sample at $2\Theta = \theta_a = 0$,

$$P = L\begin{pmatrix}\cos\psi_n \\ \sin\psi_n \\ 0\end{pmatrix}$$

The multi-analyzer stage is rotated clockwise about the point (L, 0, 0) by θ_a arriving at point P″. This can be evaluated by translating the rotation axis to the origin (P → P′), rotating, then translating back.

$$P' = L\begin{pmatrix}\cos\psi_n - 1 \\ \sin\psi_n \\ 0\end{pmatrix}$$

Rotate by θ_a: $$L\begin{pmatrix}\cos\theta_a & \sin\theta_a & 0 \\ -\sin\theta_a & \cos\theta_a & 0 \\ 0 & 0 & 1\end{pmatrix}\begin{pmatrix}\cos\psi_n - 1 \\ \sin\psi_n \\ 0\end{pmatrix} = L\begin{pmatrix}\cos\theta_a(\cos\psi_n - 1) + \sin\theta_a\sin\psi_n \\ -\sin\theta_a(\cos\psi_n - 1) + \cos\theta_a\sin\psi_n \\ 0\end{pmatrix}$$

Translate back: $$P'' = L\begin{pmatrix}\cos\theta_a\cos\psi_n - \cos\theta_a + \sin\theta_a\sin\psi_n + 1 \\ -\sin\theta_a\cos\psi_n + \sin\theta_a + \cos\theta_a\sin\psi_n \\ 0\end{pmatrix}$$

The line in the plane z = 0 through P″ has slope $\tan(\psi_n - \theta_a)$ so is $y = \tan(\psi_n - \theta_a)\,x + c$

$$c = y - \tan(\psi_n - \theta_a)\,x$$

$$c = L\big(-\sin\theta_a \cos\psi_n + \sin\theta_a + \cos\theta_a \sin\psi_n - \tan(\psi_n - \theta_a)\,(\cos\theta_a \cos\psi_n - \cos\theta_a + \sin\theta_a \sin\psi_n + 1)\big)$$

The new distance from the sample to the crystal L_n along $\begin{pmatrix}\cos\psi_n\\ \sin\psi_n\\ 0\end{pmatrix}$ intersects the crystal at

$$y = L_n \sin\psi_n = L_n \tan(\psi_n - \theta_a)\cos\psi_n + c$$

$$c = L_n(\sin\psi_n - \tan(\psi_n - \theta_a)\cos\psi_n)$$

$$L_n = \frac{L\big(-\sin\theta_a \cos\psi_n + \sin\theta_a + \cos\theta_a \sin\psi_n - \tan(\psi_n - \theta_a)\,(\cos\theta_a \cos\psi_n - \cos\theta_a + \sin\theta_a \sin\psi_n + 1)\big)}{(\sin\psi_n - \tan(\psi_n - \theta_a)\cos\psi_n)}$$

$$L_n = L(\tan(\theta_a/2)\sin\psi_n + \cos\psi_n) = L(\sin\psi_n - \sin(\psi_n - \theta_a))/\sin\theta_a$$

S2. Additional correction

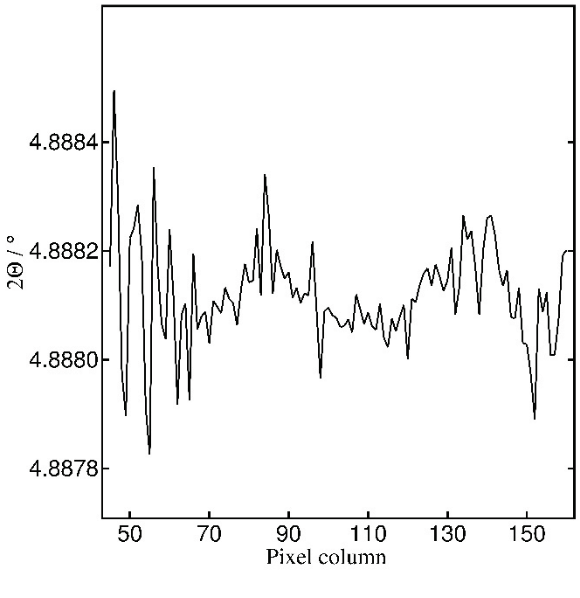

Figure S2 By including a correction to the axial position of the detected photon on the detector, z_d, of $(L + L2)\sin R_y \cos 2\Theta$ by rotation of the diffractometer by 30 μrad about the vertical axis (R_y) the trend in position of the 100 peak across the detector seen in Fig. 8c can be essentially removed, (average position 4.88812(10)°).

S3. Results of single peak fits

Table S1 Results of fitting to the LaB_6 100 and 654 peaks, using the *more_accurate_Voigt* function in Topas, with *Full_Axial_Model* asymmetry for 100, (refining the apparent size of the axial acceptance, 0.895 mm source size, 1.2 mm beam width, and source-to-sample and sample-to-detector distances of 42200 mm and 800 mm, respectively), or with one or two *exp_conv_const* exponential contributions (labeled *E*). The nine channels were summed in various ways. The equivalent axial aperture for channel 4, mounted on the rotation axis of the multi-analyzer stage, is indicated.

Summing condition	Axial range; Relative intensity; R_{exp}	Position / °	Voigt FWHM / °×10^{-3}	Asymmetry / mm and/or *exp_conv_const*	Correlation / % between position and asymmetry	R_{wp},, GOF
Uncorrected, 4mm axial aperture	4 mm 100% 2.58	4.887372(10)	2.72(3)			12.89, 5.00
		4.888179(5)	1.68(2)	4.29(1)	83	3.72, 1.45
		4.888170(8)	1.95(2)	*E* -0.00600(6)	-87	4.95, 1.92
		4.888637(13)	1.52(2)	*E* -0.00478(114)<br>*E* -0.00447(121)	81<br>84	3.86, 1.50
Corrected, 4mm axial aperture	4 mm 100% 2.57	4.887880(3)	2.15(1)			4.27, 1.65
		4.888217(13)	1.87(2)	2.57(6)	98	3.67, 1.42
		4.888257(8)	1.88(1)	*E* -0.00270(6)	-96	3.46,1.34
		4.887963(6)	1.45(2)	*E* -0.00339(5)<br>*E* 0.00274(6)	-15<br>-58	2.71, 1.05
Corrected, intrinsic broadening ≤ 002°	2.58 mm 74% 3.02	4.887871(3)	2.02(1)			4.60, 1.52
		4.888202(12)	1.73(2)	2.55(6)	98	3.87, 1.28
		4.888233(9)	1.76(1)	*E* -0.00261(7)	-97	3.66, 1.21
		4.887962(6)	1.30(2)	*E* -0.00330(5)<br>*E* 0.00258(7)	-12<br>-60	3.02, 1.00
Corrected, intrinsic broadening ≤ 0015°	1.89 mm 55% 3.51	4.887872(3)	1.95(1)			5.05, 1.44
		4.888201(12)	1.64(2)	2.55(6)	98	4.18, 1.19
		4.888245(8)	1.65(1)	*E* -0.00270(6)	-96	3.96, 1.13
		4.887969(6)	1.18(2)	*E* -0.00330(6)<br>*E* 0.00254(7)	-11<br>-60	3.33, 0.95
Corrected, intrinsic broadening	1.20 mm 37% 4.30	4.887873(3)	1.89(1)			5.75, 1.34
		4.888203(12)	1.55(3)	2.55(6)	97	4.78, 1.11
		4.888241(9)	1.58(2)	*E* -0.00267(7)	-96	4.61, 1.07

≤ 001°		4.887976(8)	1.11(3)	*E* -0.00322(7)<br>*E* 0.00240(8)	-7<br>-63	4.14, 0.97
Corrected, intrinsic broadening ≤ 0005°	0.52 mm<br>18%<br>6.17	4.887875(4)	1.86(1)			6.83, 1.11
		4.888203(15)	1.52(3)	2.55(7)	97	6.02, 0.98
		4.888232(12)	1.56(2)	*E* -0.00259(9)	-96	5.90, 0.96
		4.887973(10)	1.07(4)	*E* -0.00318(9)<br>*E* 0.00240(11)	-9<br>-61	5.51, 0.90
All	20 mm<br>510%<br>1.14	4.887924(16)	4.97(5)			11.76, 10.32
		4.888577(157)	4.65(13)	3.73(50)	99	11.71, 10.29
		4.888827(57)	4.43(7)	*E* -0.00660(43)	-96	11.34,9.97
		4.887935(12)	1.92(4)	*E* -0.0112(1)<br>*E* 0.0111(1)	-35<br>-33	4.95, 4.36
Uncorrected, 4mm axial aperture	4 mm<br>100%<br>12.86	43.94950(5)	7.5(3)			14.94, 1.16
		43.94947(13)	5.6(5)	*E* -0.0146(24)<br>*E* 0.0146(24)	-23<br>-19	12.63, 0.99
Corrected, 4mm axial aperture	4 mm<br>100%<br>12.86	43.94941(5)	7.4(3)			14.82, 1.15
		43.94930(12)	5.5(5)	*E* -0.0144(22)<br>*E* 0.0151(22)	-27<br>-17	12.70, 0.99
Corrected, intrinsic broadening ≤ 002°	20 mm<br>465%<br>5.86	43.94944(3)	7.3(2)			9.99, 1.70
		43.94949(7)	5.6(3)	*E* -0.0138(12)<br>*E* 0.0131(13)		6.81, 1.16
Corrected, intrinsic broadening ≤ 001°	12.6 mm<br>302%<br>7.41	43.94942(4)	7.2(2)			11.08, 1.50
		43.94957(8)	5.6(3)	*E* -0.0141(13)<br>*E* 0.0126(14)	-3<br>-42	7.99, 1.08

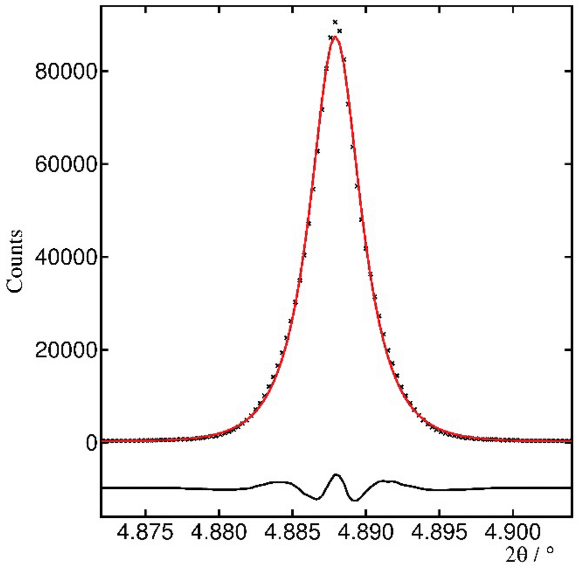

Figure S3 Fit to the 100 peak using all the angle-corrected counts across the detector. The peak is modeled with a Voigt and two (essentially symmetric) exponential contributions (see Table S1). The envelope of 1044 individual patterns shown in Fig. 7 indicates what the peak shape would be without the correction of the angular scale.

S4. Low-angle peaks

Equation 11 allows $\cos(2\Theta - \theta_a)$ to be calculated as a function of φ for any angle of diffraction from the sample, 2θ. The value of 2Θ at which the diffracted beam is transmitted by the analyzer crystal is obtained by taking $\cos^{-1}$ of the right-hand side of equation 11 and adding θa. However, if $(2\Theta - \theta a)$ falls below zero (when $2\Theta < \theta a$), the inverse cosine function still returns a positive value for $(2\Theta - \theta a)$, because $\cos(\theta) = \cos(-\theta)$, whereas the correct value for $(2\Theta - \theta a)$ is the negative of the value returned. The situation is illustrated in Fig S4.

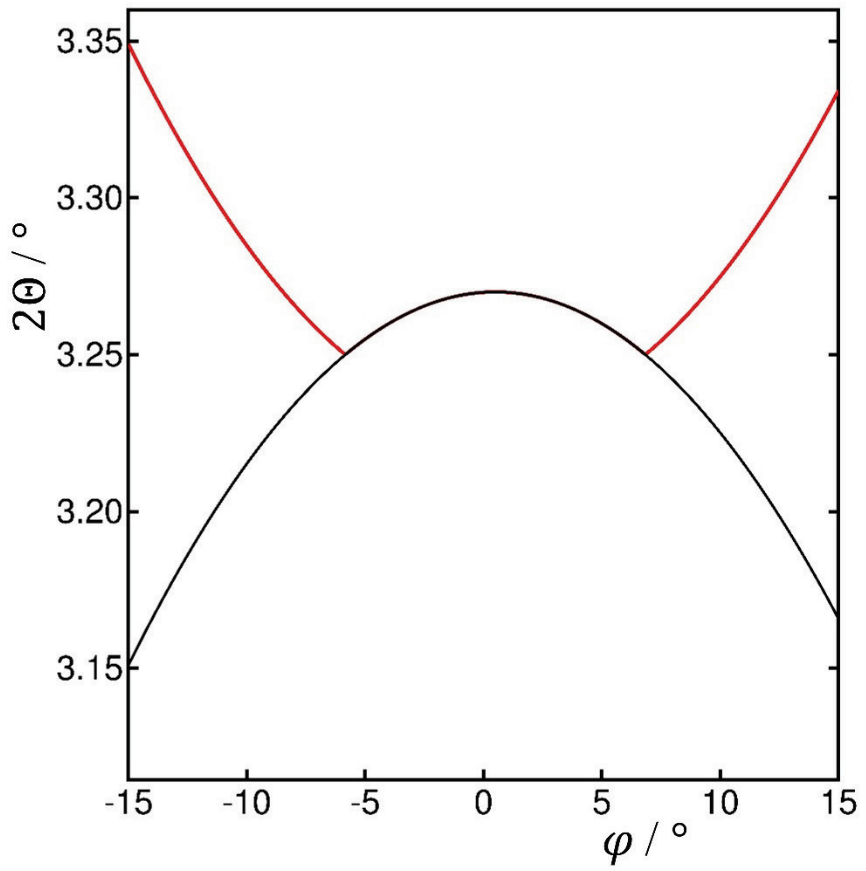

Figure S4 (*Black*) Correct calculated diffractometer arm angle, 2Θ, for a diffraction peak at 2θ = 3.27°, as a function of φ, with $\theta_a = 3.25°$ and $\vartheta_x = 0.5°$; (*red*) the result computed taking $\cos^{-1}$ of the right-hand side of equation 11 and adding θa. Once the value of $2\Theta - \theta_a$ falls below 0, the inverse cosine function yields a positive value when the correct answer is the negative of the value returned. The problem only occurs at angles near or below θ_a. (The red and black curves coincide in the central region).

The solution is to take the negative of the value for (2Θ – θa) returned via equation 11 when appropriate. The problem is to know when to take the positive and when to take the negative value of the solution. It is evident in Fig. S4 that the black (correct) curve's second derivative is negative, as the rate of change in the 2Θ angle for the transmission of the diffracted radiation increases with increasing φ, a point also noted in the main text with regard to the intrinsic broadening of peaks with distance from the centerline of the detector. By contrast, the red curve therefore has a positive value of the second derivative. It is possible to estimate numerically the second derivative of a function as $(f(x+h) - 2f(x) + f(x-h))/h^2$. Thus at each value of φ it is a straightforward matter to obtain the second derivative by evaluating $2\Theta - \theta_a$ at three points, φ and $\varphi \pm \delta$, where δ is small, (e.g. 10^{-10}). Depending on the sign of the second derivative,

so the correct value of $2\Theta - \theta_a$ can be chosen. The Topas input file can be modified to take this into account if necessary to deal with low-angle peaks (see below).
Similar reasoning holds for the correction of the data back to the true 2θ scale diffracted by the sample.

Topas input file modification to take into account low-angle data if required

```
fn dd(om, fx, fy) {

def xx = Sin(fx) Sin(fy) Cos(2 Th) - Cos(fx) Sin(2 Th) Cos(om) ;
def yy = Cos(fx) Cos(2 Th) + Sin(fx) Sin(fy) Sin(2 Th) Cos(om) ;
def zz = Sin(fx) Cos(fy) Sin(om) Sin(2 Th) - Sin(alpha) ;

def aq = xx^2 + yy^2 ;
def bq = -2 zz xx ;
def cq = zz^2 - yy^2 ;

def cosTT = (-bq + Sqrt(bq^2 - 4 aq cq)) / (2 aq) ;

def TT =  ArcCos(cosTT) ;

/* For low angle, with 2 Th near alpha or below, TT should be negative, but the ArcCos function
reverses when cosTT becomes 1.
By calculating numerically the second derivative of TT with respect to om, we can see whether TT
should be positive or negative.  Next few lines calculate the second derivative.  *\

def xx1 = Sin(fx) Sin(fy) Cos(2 Th) - Cos(fx) Sin(2 Th) Cos(om-1e10) ;
def yy1 = Cos(fx) Cos(2 Th) + Sin(fx) Sin(fy) Sin(2 Th) Cos(om-1e10) ;
def zz1 = Sin(fx) Cos(fy) Sin(om-1e10) Sin(2 Th) - Sin(alpha) ;

def aq1 = xx1^2 + yy1^2 ;
def bq1 = -2 zz1 xx1 ;
def cq1 = zz1^2 - yy1^2 ;

def xx3 = Sin(fx) Sin(fy) Cos(2 Th) - Cos(fx) Sin(2 Th) Cos(om+1e10) ;
def yy3 = Cos(fx) Cos(2 Th) + Sin(fx) Sin(fy) Sin(2 Th) Cos(om+1e10) ;
def zz3 = Sin(fx) Cos(fy) Sin(om+1e10) Sin(2 Th) - Sin(alpha) ;
```

```
def aq3 = xx3^2 + yy3^2 ;
def bq3 = -2 zz3 xx3 ;
def cq3 = zz3^2 - yy3^2 ;

def cosTT1 = (-bq1 + Sqrt(bq1^2 - 4 aq1 cq1)) / (2 aq1) ;
def cosTT3 = (-bq3 + Sqrt(bq3^2 - 4 aq3 cq3)) / (2 aq3) ;

def TT1 = ArcCos(cosTT1) ;
def TT3 = ArcCos(cosTT3) ;

'Second derivative
def  TTfpp = TT1 - 2 TT + TT3 ;

TT = If(TTfpp < 0, TT, -TT) ;

def Delta = (TT + alpha - 2 Th) ;   ' Delta is in radians

return  Delta ;
 }
```
